# Supplementary figures and images for: APE1 Promotes Pancreatic Cancer Proliferation through GFRα1/Src/ERK Axis-Cascade Signaling in Response to GDNF
Source: Int J Mol Sci. 2020 May 19;21(10):3586. doi: 10.3390/ijms21103586 (PMC7279477; doi:10.3390/ijms21103586)

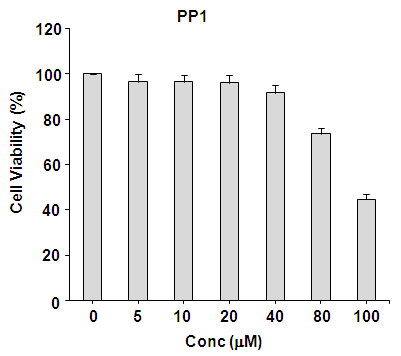

Supplement: Supplementary file 1 [file ijms-21-03586-s001.zip › ijms-792973-supplementary.tif]
